# Personal Beauty

**Published:** 1892-02

**Authors:** 


### PERSONAL BEAUTY.

Several English medical journals have recently called attention to a fact, sustained by common observation, that the young women of the present day are better developed physically, taller, plumper, stronger, and healthier than the young women of 50, 100, 150, or 200 years ago, and that in this continuous and perceptible improvement of condition and aspect there are no steps backward. One journal has called attention to the circumstances that whereas a century or two ago a handsome woman inspired sentiments of such admiring curiosity that her arrival or departure drew vast crowds and rewarded the patient waiting of hundreds, beauty of the same sort is so general now-a-days as to evoke no ripple of excitement. It is no longer so rare that it commands peculiar attention. Again, the portraits of women of acknowledged loveliness preserved in many houses and galleries, or public buildings, and wondered at for many years, cannot stand comparison with many living countenances whose good looks are so little beyond the ordinary as to inspire neither poet, painter, composer, nor sculptor with a subject for rhapsody in sonnet, on canvass, by lyric, or in marble. But the change to which English writers on hygiene allude in scientific phrase and without passion or emotion, is not limited to their field of personal observation. The tendency of women to grow handsomer, and of women no longer young to remain beautiful, has its manifestation in Canada, too, and in ample and undisguisable degree. Generally speaking, the proportion of handsome women is larger than it used to be, and uncomeliness is diminished correspondingly. The true causes of this are probably two:

1. Improved health, the result of a more scientific mode of life, better hygienic conditions, larger latitude in outdoor exercise, better nutrition, better physical culture, and softening and equalizing of the climate; and 2. Better taste in dress, the introduction of new and becoming methods of attire, improved style, a greater variety of fabrics and colors, and such cheapening of materials for a girl's wear as to bring them within the reach of all.—*Toronto Truth*.

